# Comprehensive Analysis of Hepatitis B Virus Promoter Region Mutations

**DOI:** 10.3390/v10110603

**Published:** 2018-11-01

**Authors:** Vanessa Meier-Stephenson, William T. R. Bremner, Chimone S. Dalton, Guido van Marle, Carla S. Coffin, Trushar R. Patel

**Affiliations:** 1 Department of Microbiology, Immunology and Infectious Diseases, Cumming, School of Medicine, University of Calgary, Calgary, AB T2N 1N4, Canada; vmeierstephenson@gmail.com (V.M.-S.); vanmarle@ucalgary.ca (G.v.M.); cscoffin@ucalgary.ca (C.S.C.); 2 Alberta RNA Research & Training Institute, Department of Chemistry & Biochemistry, University of Lethbridge, Lethbridge, Alberta, T1K 3M4, Canada; 3 Department of Ecosystem & Public Health, Faculty of Veterinary Medicine, University of Calgary, Calgary, AB T2N 4N1, Canada; william.bremner1@ucalgary.ca (W.T.R.B.); csdalton@ucalgary.ca (C.S.D.); 4 Liver Unit, Division of Gastroenterology and Hepatology, Department of Medicine, Calgary, AB T2N 4Z6, Canada; 5 DiscoveryLab, Faculty of Medicine & Dentistry, Li Ka Shing Institute of Virology, University of Alberta, Edmonton, AB T6G 2H7, Canada

**Keywords:** Hepatitis B virus (HBV), cccDNA, basal core promoter, X promoter, single nucleotide polymorphisms, logo analyses, genotype alignments

## Abstract

Over 250 million people are infected chronically with hepatitis B virus (HBV), the leading cause of liver cancer worldwide. HBV persists, due, in part, to its compact, stable minichromosome, the covalently-closed, circular DNA (cccDNA), which resides in the hepatocytes’ nuclei. Current therapies target downstream replication products, however, a true virological cure will require targeting the cccDNA. Finding targets on such a small, compact genome is challenging. For HBV, to remain replication-competent, it needs to maintain nucleotide fidelity in key regions, such as the promoter regions, to ensure that it can continue to utilize the necessary host proteins. HBVdb (HBV database) is a repository of HBV sequences spanning all genotypes (A–H) amplified from clinical samples, and hence implying an extensive collection of replication-competent viruses. Here, we analyzed the HBV sequences from HBVdb using bioinformatics tools to comprehensively assess the HBV core and X promoter regions amongst the nearly 70,000 HBV sequences for highly-conserved nucleotides and variant frequencies. Notably, there is a high degree of nucleotide conservation within specific segments of these promoter regions highlighting their importance in potential host protein-viral interactions and thus the virus’ viability. Such findings may have key implications for designing antivirals to target these areas.

## 1. Introduction

Hepatitis B virus (HBV) is a partially double-stranded DNA virus belonging to the *Hepadnaviridae* family that chronically infects over 250 million people worldwide [1]. Infection is associated with 25–40% lifetime risk of severe liver disease, including cirrhosis, liver failure, and hepatocellular carcinoma (HCC) [1]. There is no cure for HBV, and while current nucleos(t)ide analog treatments act to suppress viral replication, virological relapses often occur once therapy is stopped [2,3,4,5]. This is due to the persistence of the virus’ minichromosome or covalently closed circular DNA (cccDNA). A therapy against chronic HBV will therefore need to target cccDNA [6,7,8,9]. The search for new targets in this compact, challenging the virus has led some researchers to consider targeting the involved host proteins [10,11]. While promising, this approach may harbor some unwanted off-target effects on the host. An alternative strategy is to analyze the reciprocal interaction for new therapeutic targets, i.e., the viral region(s) that are recognized by the host proteins.

Gene promoter regions are essential sites in DNA recognized by proteins for the downstream processes of replication and transcription [12,13]. Mutations in promoter regions, as a generalization, can often lead to down or up-regulation of genes that may lead to malignancies [14,15,16,17,18]. For example, in gastric adenocarcinomas, specific mutations in the *AKT1* promoter region have been identified from clinical samples and confirmed in vitro to upregulate the Akt/PKB (protein kinase B) pathway, a central pathway for cancer cell proliferation [17,18]. Additionally, *TERT* gene promoter mutations lead to the upregulation of hTERT (human telomerase reverse transcriptase), a key step in cell immortalization, and has been found in over 40% of cutaneous melanoma, effectively acting as an independent prognostic factor for disease [19,20]. Promoter region mutations that occur in the context of properly functioning DNA proofreading mechanisms are corrected or naturally-selected out if not beneficial to the organism, thus maintaining the genome and resulting in conserved sequences.

Similar to the genome organization in higher organisms, pathogens such as viruses and bacteria also contain promoter sites in their genome. However, unlike most living organisms, viruses require the host-machinery to survive. Upon infecting a host cell, viruses manipulate and utilize host proteins to enable its replication and survival. Host proteins are similarly recruited to viral promoter regions for the analogous replicative processes. For example, HIV-1 recruits Sp1 to its COMMD1 (COMM domain-containing protein 1) promoter region to regulate transcription [21]; and in human papillomavirus (HPV), Sp1, AP1, and NF1 are recruited to early promoters to initiate transcription [22,23,24]. Thus, for effective infection, those regions that interact with the host proteins would also need to be highly conserved.

**Figure 1:**
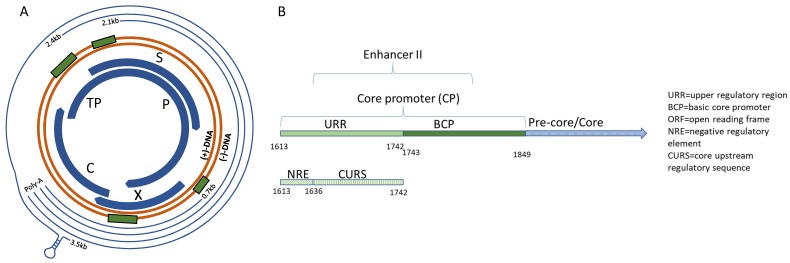
Schematic of the HBV genome. (**A**) From the (−)-strand DNA, overlapping reading frames code for gene segments C, P, S, and X. Transcripts are depicted by the outer thin lines and ending in the poly-A adenylation sequence. The X and Core promoter regions are highlighted by green rectangles preceding the corresponding gene. (**B**) Core promoter schematic.

Hepatitis B virus’ stable genome, the cccDNA, comprises of four genes, C, S, P, and X (Figure 1A). The genes are arranged into four overlapping reading frames, the transcripts of which are controlled by four different promoter regions—C, X, PreS1, and PreS2, again overlapping with a segment of the preceding gene [25,26,27]. As such, HBV has one of the smallest genomes, comprising only 3.2 kb. During chronic infection, up to 10^12^ virions can be produced per day, which in combination with the highly error-prone HBV polymerase, can give rise to up to 10^10^–10^11^ point mutations daily generating innumerable viral quasispecies with varying degrees of fitness [28]. Viruses with reduced fitness would be selected out over time, leaving the most stable, replication-competent viruses to be recovered from clinical infection.

In 2012, researchers from France started an international repository of sequenced clinical HBV specimens. The database (HBVdb) is open-access [29] and currently has nearly 70 000 reference genomes [30]. Given that these genomes are based on clinical specimens, the repository thus represents an extensive database of *replication-competent* viruses.

In this work, the HBV core and X promoter regions were analyzed across this extensive HBV repository for mutational frequencies in all known genotypes, with the hypothesis that regions exhibiting the highest nucleotide conservation are more likely to be critical in the host protein-viral DNA interaction. Indeed, we find that there is considerable nucleotide conservation throughout the promoter regions, amongst and between genotypes. These conserved sites highlight specific sub-regions as likely essential for maintaining a host protein interaction, and thus may make reasonable targets for antiviral therapy.

## 2. Materials and Methods

HBV sequences were sourced from the publicly accessible HBV database [29], where genotypes A-H are collected and categorized based on the literature-accepted >8% difference across the whole genome [30]. This study included 840 whole-genome sequences of genotype A; 1700 genotype B; 2153 genotype C; 948 genotype D; 255 genotype E; 248 genotype F; 39 genotype G; and 26 genotype H. Alignments were imported into Geneious 10.1.3 [31] in FASTA format for downstream analysis. The consensus sequence for the alignment of all available whole genome HBV sequences was extracted and annotated for promoter binding regions previously identified in the core promoter region [32,33,34]. These annotations included C/EBP-like, HNF4, HNF3, SP1, and TBP binding sites. The same annotations were further applied to individual genotype alignments (HBV genotypes A–H) and extracted as separate alignments. Owing to differences in the alignments derived from the HBV database, each genotype required separate annotation. The genome positions of the relevant binding sites for each genotype are summarized in a table format (Supplementary Table S1). Genotype alignments of promoter binding sites were then converted to sequence logos using WebLogo [35,36] and manually stacked for comparison. Variant frequency tables were additionally generated for each alignment converted to a sequence logo using a 0.05% frequency threshold using the “find SNP/variants” function of Geneious and are included in the supplementary material (Supplementary Table S1). Poly-mutational analyses of known high-risk mutations per genotype, including the Fisher exact test were performed using Microsoft Excel.

## 3. Results

### 3.1. X Gene Promoter Region

**Figure 2:**
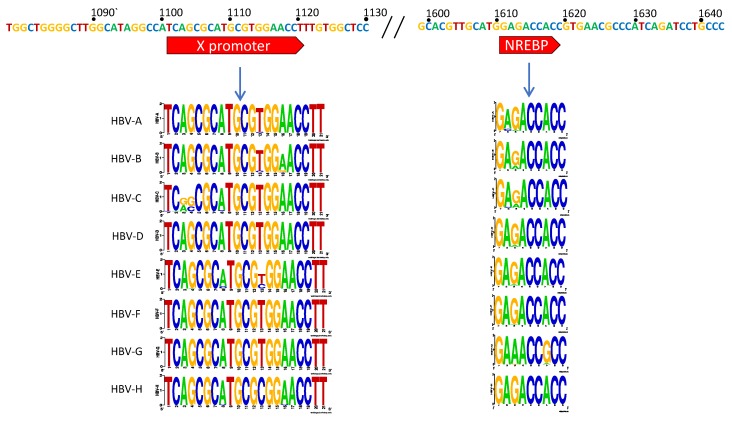

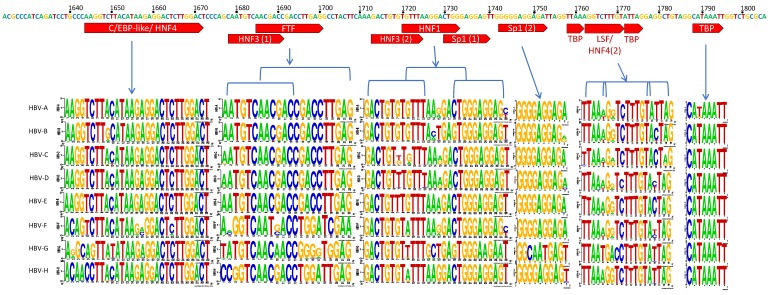
HBV X and Core promoter regions. Upper bar represents the consensus sequence across all genotypes. Highlighted segments refer to previously identified binding sites for various host proteins. Stacked LOGO sequences are displayed per genotype to give a pictorial representation of conserved nucleotides and variants. The complete consensus for the core promoter (nts 1613–1849) is included in the Supplementary Figure S1.

The X gene promoter region extends from nts 1101–1121 and aides the production of the transcript for the small protein HBx (17 kDa) [37,38,39]. This protein does not share homology with any other known gene and has been found to play roles in upregulation of the core promoter and thus HBV replication as well as being central to the pathogenesis of HBV-induced hepatocellular carcinoma (HCC) [39,40,41,42,43,44,45]. The host protein, nuclear respiratory factor 1 (NRF-1), has been shown to be responsible for X promoter binding and transcription initiation [39]. In our analyses, we note two nucleotide variants at positions 1103 and 1104 of the genotype C sequence logo are unique compared to the other genotypes (Figure 2A). Additionally, nt 1113 had some variance between C and T. All other nucleotides had high levels of conservation, providing the consensus sequence: TCAGCGCATGCGTGGAACCTT for this region.

### 3.2. C Gene/HBV Core Promoter Region

The Core promoter (CP) spans nts 1611–1847 and can be subdivided into the upper regulatory region (URR) and basic core promoter (BCP) (Figure 1B) [37]. Within the URR is a segment known as the negative regulatory element (NRE) which, when bound by the NRE-binding protein, can suppress the core promoter activity by approximately 10–20-fold [46]. Isolates in the HBV database show a high degree of conservation throughout this region, with the exception of nt 1613 where there were G to A variations amongst all genotypes. Also, genotype G was predominantly A at nt 1617, while all others were G (Figure 2A). The core upstream regulatory sequence (CURS, nts 1634–1740), contains positively regulating regions, including the hepatocyte-enriched transcription factor, HNF-4, which can activate the core promoter approximately 20-fold [47]. This segment has been identified to span approximately nts 1648–1672/1682 [32,34,47]. In a similar overlapping region, CAAT enhancer-binding protein (C/EBP) has also been shown to bind and increase the promoter activity in a dose-dependent manner [48]. When this region is analyzed, there appears to be high conservation across genotypes A–E, while several variants are present through nts 1645–1649 of genotypes F, G, and H (Figure 2A).

Another hepatocyte nuclear factor, HNF-3, has three binding sites: One in Enhancer I (nts 1120–1130—further upstream from the promoter) and two in the CURS region (CURS/Enhancer II; nts 1679–1690 and 1713–1723). Binding of HNF-3 to Enhancer I region increases the promoter activity by ~15 fold [49,50], whereas to CURS region increases promoter activity in a dose-dependent manner [49,51]. The first of these two CURS segments show high conservation across genotypes A to E, but has significant variations noted for the equivalent region in genotypes F to H. The second region is nearly completely conserved throughout except for nts 1719 and 1721, with two possible variants present at each site, G and T for nt 1719, and G and A for nt 1721 (Figure 2B). Adjacent to this region is an HFN1 binding element (nts 1721–1734), that can also greatly upregulate replication. In a cohort of patients with severe HBV-related liver disease, clinical variants were identified containing two HFN1 binding regions [32]. Subsequent primer analyses studies showed that duplication of the HFN1 binding region is enough to compensate for loss of Sp1 binding regions [32]. Across genotypes, there is general consensus, but with much more variation than in some of the other binding regions. In particular, nt 1721 has a G to A variability in genotypes C, F, and H, and a G to T variability in genotype G. Similarly, nts 1726–1727 have the greatest degree of variation, even within the genotype (Figure 2B).

In the basic core promoter (BCP), the minimal essential sequence is composed of a 108-bp fragment (nts 1740–1847) [37]. Within this fragment is the direct repeat 1 (DR1), which is required for HBV polymerase binding and reverse transcription [37,52]. The BCP also contains two Specificity protein 1 (Sp1) binding sites that are critical for the transcription of mRNA from the core promoter [53,54]. The first of these Sp1 regions has a nucleotide variant present at nt 1740 in genotypes A and F, where C appears in approximately 66% of the genomes in the database (genotypes A to F), as opposed to T, which is nearly entirely conserved at this position across all other genotypes (Figure 2B). In the second Sp1 binding site, there is essentially 100% agreement across nts 1743–1751 in all but one genotype, genotype G, where there is a notable disruption of the G rich region (nts 1745–1748). The terminal nucleotide of this binding region has a notably different variant profile in genotypes G and H, where T is present in place of A. Further downstream from the Sp1 sites is a region containing a second HNF4 binding site flanked by two TATA-like binding protein (TBP) segments, nts 1758–1776 [32,33]. The stacked Logo representation generated for this region suggests this to be the least conserved amongst those assessed in this study, with notable variants present at three positions, nts 1762, 1764, and 1773. Apart from genotype G, all genotypes share high frequency variants at both nt 1762 (A->T) and 1764 (G->A). While genotypes F, B, D, and G had nucleotide variants visible in the gene logo at nt 1773 (T->C) (Figure 2B).

### 3.3. High-Risk HCC Mutations

**Figure 3:**
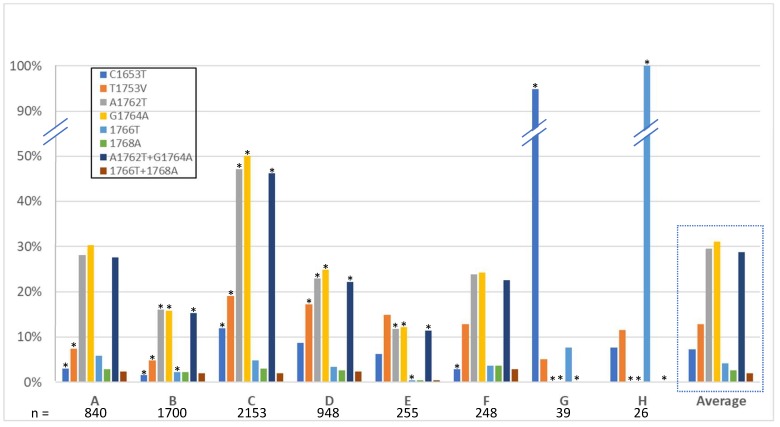
High-risk HCC mutations by genotype. The average of each mutation across all genotypes is outlined by a dashed box. Asterixis refer to significance based on average value.

The basal core promoter is the region harboring the greatest number of high-risk HCC mutations. With links of these mutations to HCC development [55] and HCC development to genotype [56], it follows that there is likely a propensity of these mutations to be found more commonly in certain genotypes. Indeed, the literature suggests a greater propensity for genotype C compared to genotype B to develop HCC when compared in large Asian cohorts [57,58,59]. A similar propensity was seen in a smaller, but genotypically diverse longitudinal study on Alaskan natives, whereby genotype C had the greatest risk of HCC development, followed distantly by F, then A [58,60]. Single and multiple-mutational frequencies by genotype are shown in Figure 3. Genotype C sequences analyzed contain the most mutations overall, with a high percentage of A1762T and G1764A mutants, nearly all of those as the double-mutant. Genotypes B and E have significantly less than the average. Genotypes G and H appear to have consensus mutations C1653T and 1766T, respectively, throughout all the available sequences from the database, while little to no other mutations are present.

## 4. Discussion

Essential to the replication of HBV are the promoter regions of each of the open reading frames (core, preS1, preS2, and X) and enhancers I and II, which interact with host proteins to enable transcription, translation, and subsequent viral propagation. Given their key grounding point, promoter regions must maintain a degree of conservation for essential nucleotides in this interaction. Indeed, such is the case found here, where key identified binding sites within the promoter regions have an extremely high degree of sequence conservation.

### 4.1. The X Promoter

The X promoter region initiates transcription of the X gene, which creates the 154-amino acid HBx protein. HBx has been shown to enhance replication of HBV and promote its integration into host DNA [61,62,63,64]. It also has the ability to manipulate numerous intracellular processes, including DNA repair mechanisms [65], DNA methylation with subsequent downstream cell proliferation, cycling, and apoptosis [66,67], interfere with key cell signaling pathways [68,69,70] and induce tumor metastases [41,71,72]. Given HBx’s extensive implications in HBV replication and HBV-induced hepatocellular carcinoma, its promoter may be an important target of HBV therapeutic design [40,41,42,43,44,45,73]. In our analysis, apart from a few exceptions, we find highly consistent nucleotide frequencies in the X promoter region throughout all genotypes (Figure 2A).

### 4.2. Importance of the C Gene/HBV Core Promoter Region

The Core promoter region is arguably the most important of the HBV promoters as it directs transcription initiation for both the pre-core and pre-genomic RNAs. Mutations in this region would thus have direct impacts on how well the virus is able to replicate. Particular regions of interest for drug targeting would thus be at any of the host protein-binding sites noted in Figure 2, highlighting the upper sequence as the consensus across all genotypes.

There are a number of mutations in the core promoter that have been linked with increased risk of HBV-induced HCC. One of the more recognized is that of the natural double mutation in the BCP, A1762T, and G1764A. This double mutation prevents the binding of several nuclear receptors while maintaining the binding of HNF4. Simultaneously, this creates a binding site for the transcription factor HNF1 and subsequently affects two amino acids of the HBx protein, the effects of both combinations greatly enhance viral replication [43,74,75,76]. The double mutant is also associated with decreased production of HBeAg [77,78]. Should a therapeutic be designed to target these regions with high-risk HCC mutations, the varied consensus should thus be taken into consideration, including the neighboring nucleotides that enable alternate binding.

Sp1 binding sites are also critical to the transcription of mRNA from the core promoter [53,54]. In particular, we note the two previously identified Sp1 binding sites and highlight their highly G-rich nature. This region alone harbors a >99% conservation across all but genotype G, which has three fewer G’s in this segment (nts 1745, 1746, and 1748) as well as other unique features.

#### 4.2.1. Genotype G has Many Unique Core Promoter Features

Genotype G, represented by 39 sequences in the HBV database, has a C1653T nucleotide predominance in the early promoter, possibly creating an additional TATA-binding site (Figure 2). As well, it has much less G-rich Sp1 binding region in the pre-core promoter than the other genotypes, potentially influencing binding of Sp1 in this region (Figure 2). Additionally, it contains a 36-bp insert at the 5’ end of the core gene near the ε encapsidation signal [79]. This extension results in altered base-pairing, but instead of decreasing efficiency of translation initiation and/or RNA packaging, researchers show that the virus is still replication competent with greatly enhanced core protein production [79]. When the “extension” was inserted into a similar genotype, A plasmid, the corresponding replication markers were also enhanced, supporting the independence of this insertion to enable viral competence [79]. Interestingly, when found clinically, genotype G is nearly always co-infected with genotype A, suggesting a degree of necessity of this genotype for infection, if not maintenance [80,81,82]. However, given that the occasional monoinfection with genotype G has been described [81,83,84], other explanations may be plausible.

Genotype G does not produce e antigen (HBeAg), a protein normally translated from the longer 3.5-kb pre-core mRNA, due to the presence of two stop codons in the pre-core region halting its production [85,86]. While HBeAg’s role in HBV infection remains incompletely elucidated, it has been shown to be heavily linked with numerous immunologic processes and likely contributes to the immune evasion necessary to establish chronic infection [79,87,88,89]. Some non-G strains go on to develop an e-antigen negative status over time, but initial phases would otherwise have this. Genotype G is thus likely dependent on other genotypes to establish persistence, but once established, can eventually out-compete its supporting genotype (i.e., genotype A) to create a monoinfection state in a host [86,90,91,92].

Studying genotype G’s many unique features may give further insight into HBV’s replication process. Despite HBeAg production and a lack of a key G-rich pre-core promoter region, genotype G would appear to have further modifications and symbioses that make this strain sustainable.

#### 4.2.2. Other Core Promoter Interactions

Additional factors have been found to influence HBV transcription, and thus inferred interaction with the BCP. These include testicular orphan receptor 4 (TR4) and chicken ovalbumin upstream promoter transcription factor (COUP-TF), which have been shown to competitively inhibit other factors of the core promoter in vitro through direct binding at regions described above, but it is unclear as to the degree this influences the in vivo situation [34,38,93]. Additional factors, peroxisome proliferator-activated receptors (PPAR) and retinoid X receptors (RXR), also appear to influence transcription through the core promoter, though it would appear to be more of an indirect influence on HNF4 and not directly with the promoter nucleotides themselves [34,94].

### 4.3. High-Risk HCC Mutations

Studies employing clinical isolates with associated clinical data, including disease severity and presence of HCC have linked numerous mutations with that of a greater risk of developing cancer [37,55,95]. As well, the link between HCC risk and that of genotype have also been made, i.e., per the Taiwanese REVEAL study, genotype C has a higher chance of developing HCC, with a hazard ratio of 2.99 when the double-mutation A1762T/G1764A is present [56]. While the database lacks clinical outcome data, we were able to quantify relationships between strongly-correlated high-risk HCC mutations and genotype (Figure 3, Supplementary Table S2). The database, a representation of the currently available clinical isolates, is consistent with prior findings, showing a high prevalence of mutations, including the double-mutant, in genotype C. It was also notable that there was a high rate of the triple-mutation T1753V/A1762T/G1764A in genotype C. Further, the analyses also revealed significantly fewer mutations appearing in genotypes B and E.

### 4.4. Study Limitations

One limitation of the study is the reliability of the sequences deposited in the HBVdb. While the database has control measures in place to provide quality annotations and sequence information, its sequences are provided by researchers with varying sequencing expertise. Additionally, while consensus sequences are typically compiled from many reads of a single piece of DNA, our attempt at creating a “consensus” will be skewed by using thousands of sequences across eight genotypes. This will inherently overlook unique genotypic features (i.e., genotype G’s 34-bp insertion) and give stronger weight to the more abundant genotypes (i.e., genotype C), and thus conclusions drawn therein will have to take such biases into consideration. Lastly, given the limited number of sequences available for genotypes G and H, largely due to epidemiologic and endemicity factors, inferences from the above analyses will also be limited.

### 4.5. Future Perspectives

Applying the findings of this work to further applications, one may be able to narrow the focus certain host protein-interactions to a more well-defined nucleotide region that persists throughout all competent viruses. Through comprehensive genotype comparisons of HBV, it may be possible to adapt or develop therapeutics for pan-genotypic applications. Additionally, mutations in host protein binding regions associated with hepatocellular carcinoma could be exploited with structural studies or binding assays to reveal unique pathogenicity features. Lastly, the approach described in this paper may be applied to investigate other viral nucleotide regions involved in host protein interactions.

## 5. Conclusions

Our frequency analyses of the HBV sequences available in the HBVdb demonstrate a high degree of conservation across the X and C promoters of all genotypes. In particular, regions with known host-protein binding have nucleotide conservation of over 99% strongly suggesting the importance of these specific nucleotides in the interaction with the host cell. These host-binding regions could thus be capitalized on, narrowing the focus of a study interaction, and importantly aiding the study of how the interaction may differ from that of the host protein’s usual target(s). Further, it can provide insights into regions in the HBV genome that can be targeted for a selective yet pan-genotypic rational drug design for treatment of HBV infection.

## Supplementary Materials

The following are available online at http://www.mdpi.com/1999-4915/10/11/603/s1, Table S1: X and core promoter variant frequencies; Table S2: Single- and multiple-mutation frequencies per genotype. Supplementary Figure S1: Consensus sequence for the core promoter region.
