# Supplementary material for: Comprehensive Analysis of Hepatitis B Virus Promoter Region Mutations
Source: Viruses. 2018 Nov 1;10(11):603. doi: 10.3390/v10110603 (PMC6265984; doi:10.3390/v10110603)
Supplement: Supplementary file 1 [file viruses-10-00603-s001.zip › viruses-380141-SI/viruses-380141-supplementary-FINAL/viruses-380141-r1-supplementary.pdf]

Supplementary Table S1. Variant frequencies within the core promoter binding regions. Values reported to values 0.5%-99.5%; values between 5-95% are highlighted in green.

X promoter:	1101-1121		
Genotype	Position	Variant Frequency	Variant Nucleotide(s)
A	1113	4.40%	C
A	1113	95.40%	T
B	1117	99.40%	A
B	1117	0.60%	G
B	1116	86.00%	A
B	1116	2.60%	C
B	1116	11.40%	G
B	1113	7.70%	C
B	1113	91.60%	T
B	1108	98.00%	A
B	1108	0.70%	C
B	1108	1.10%	G
B	1107	99.10%	C
B	1107	0.90%	T
B	1103	99.30%	A
B	1103	0.70%	G
C	1113	0.70%	C
C	1113	0.70%	G
C	1113	98.40%	T
C	1108	94.30%	A
C	1108	3.80%	C
C	1108	1.00%	G
C	1108	1.00%	T
C	1105	99.30%	C
C	1105	0.50%	G
C	1104	33.70%	C
C	1104	66.00%	G
C	1103	46.90%	A
C	1103	53.00%	G
C	1102	2.00%	A
C	1102	97.40%	C
C	1101	2.30%	C
C	1101	97.60%	T
D	1117	98.60%	A
D	1117	0.50%	G
D	1117	0.50%	T
D	1108	99.30%	A

D	1108	0.50%	C
D	1101	0.60%	C
D	1101	99.30%	T
E	1113	32.00%	C
E	1113	67.60%	T
E	1110	1.60%	A
E	1110	98.00%	G
E	1108	88.00%	A
E	1108	10.40%	C
E	1108	1.60%	G
E	1104	1.60%	A
E	1104	98.40%	G
F	1117	98.80%	A
F	1117	0.80%	G
F	1108	98.80%	A
F	1108	1.00%	C
H	1116	96.20%	A
H	1116	3.80%	C
H	1108	96.20%	A
H	1108	3.80%	C

NREBP: nts 1611-1619

Genotype	Position	Variant Frequency	Variant Nucleotide(s)
A	1613	4.90%	A
A	1613	95.10%	G
A	1612	90.20%	A
A	1612	9.80%	C
B	1617	99.40%	A
B	1617	0.60%	C
B	1613	19.70%	A
B	1613	80.30%	G
B	1612	98.90%	A
B	1612	0.90%	G
C	1613	22.70%	A
C	1613	77.30%	G
D	1613	7.30%	A
D	1613	92.70%	G
E	1618	98.80%	C
E	1618	1.20%	T
E	1613	5.70%	A

E	1613	94.30%	G
F	1613	2.00%	A
F	1613	98.00%	G
G	1617	5.10%	A
G	1617	94.90%	G
G	1613	97.40%	A
G	1613	2.60%	G
H	1617	98.10%	A
H	1617	1.90%	C

C/EBP-HNF4(1): nts 1644-1672

Genotype	Maximum	Variant Frequency	Variant Nucleotide(s)
A	1659	98.90%	A
A	1659	1.00%	C
A	1653	96.80%	C
A	1653	3.20%	T
B	1645	97.10%	A
B	1645	2.70%	C
C	1665	0.60%	A
C	1665	99.40%	C
C	1661	0.60%	A
C	1661	99.40%	G
C	1659	97.00%	A
C	1659	2.90%	C
C	1658	1.00%	A
C	1658	98.70%	G
C	1655	1.60%	C
C	1655	98.30%	T
C	1653	87.80%	C
C	1653	12.20%	T
C	1652	95.70%	A
C	1652	4.30%	G
C	1647	1.50%	C
C	1647	98.50%	G
D	1665	3.00%	A
D	1665	97.00%	C
D	1661	1.30%	A
D	1661	98.70%	G
D	1659	98.60%	A
D	1659	1.40%	C

D	1655	0.50%	C
D	1655	99.50%	T
D	1653	90.90%	C
D	1653	9.10%	T
D	1652	97.50%	A
D	1652	2.50%	G
D	1647	1.90%	A
D	1647	98.10%	G
E	1659	98.40%	A
E	1659	1.60%	C
E	1653	93.50%	C
E	1653	6.50%	T
E	1652	98.80%	A
E	1652	0.80%	G
E	1649	98.80%	C
E	1649	1.20%	T
E	1648	1.20%	G
E	1648	98.40%	T
F	1665	16.10%	A
F	1665	83.70%	C
F	1659	56.30%	A
F	1659	43.50%	C
F	1658	12.10%	A
F	1658	1.20%	C
F	1658	86.30%	G
F	1655	1.60%	C
F	1655	98.20%	T
F	1653	97.00%	C
F	1653	3.00%	T
F	1648	5.20%	C
F	1648	94.80%	T
F	1646	98.60%	A
F	1646	1.20%	G
F	1645	1.20%	A
F	1645	98.00%	C
F	1645	0.80%	T
G	1659	94.90%	A
G	1659	2.60%	C
G	1659	2.60%	T
G	1653	5.10%	C
G	1653	94.90%	T

G	1652	94.90%	A
G	1652	5.10%	G
G	1649	5.10%	A
G	1649	94.90%	G
G	1648	94.90%	A
G	1648	5.10%	T
G	1647	94.90%	C
G	1647	5.10%	G
G	1645	33.30%	A
G	1645	66.70%	G
H	1670	96.20%	A
H	1670	3.80%	T
H	1661	7.70%	A
H	1661	92.30%	G
H	1658	3.80%	A
H	1658	96.20%	G
H	1653	92.30%	C
H	1653	7.70%	T
H	1652	96.20%	A
H	1652	3.80%	G
H	1647	96.20%	A
H	1647	3.80%	C

Site1: HNF3(1)-FTF: nts 1679-1700

Genotype	Maximum	Variant Frequency	Variant Nucleotide(s)
B	1688	4.20%	A
B	1688	95.60%	G
B	1687	95.70%	C
B	1687	4.30%	T
B	1683	1.00%	C
B	1683	1.20%	G
B	1683	97.80%	T
B	1680	98.90%	A
B	1680	0.90%	C
B	1679	98.90%	A
B	1679	0.90%	G
C	1688	1.40%	A
C	1688	98.60%	G
C	1680	99.00%	A
C	1680	0.90%	C

C	1679	90.80%	A
C	1679	1.30%	C
C	1679	7.70%	G
D	1694	98.00%	C
D	1694	2.00%	T
D	1679	92.90%	A
D	1679	1.90%	C
D	1679	5.10%	G
E	1693	98.80%	A
E	1693	0.80%	T
E	1691	99.20%	C
E	1691	0.80%	G
F	1700	98.80%	A
F	1700	0.80%	G
F	1699	98.80%	A
F	1699	1.20%	C
F	1697	97.20%	C
F	1697	2.40%	T
F	1695	99.20%	A
F	1695	0.80%	C
F	1694	0.80%	C
F	1694	99.20%	G
F	1693	0.80%	A
F	1693	99.20%	G
F	1692	0.80%	C
F	1692	0.80%	G
F	1692	98.20%	T
F	1689	96.60%	A
F	1689	3.40%	C
F	1688	33.70%	C
F	1688	66.10%	G
F	1687	1.20%	C
F	1687	98.40%	T
F	1685	99.00%	A
F	1685	0.80%	C
F	1684	99.00%	C
F	1684	0.80%	T
F	1682	0.80%	A
F	1682	99.20%	G
F	1681	99.00%	G
F	1681	1.00%	T

F	1680	45.00%	A
F	1680	44.40%	C
F	1680	4.00%	G
F	1680	6.70%	T
F	1679	98.60%	A
F	1679	1.40%	G
G	1697	94.90%	G
G	1697	5.10%	T
G	1695	10.30%	A
G	1695	5.10%	C
G	1695	84.60%	G
G	1694	5.10%	C
G	1694	94.90%	G
G	1693	5.10%	A
G	1693	94.90%	G
G	1688	94.90%	A
G	1688	5.10%	G
G	1679	5.10%	A
G	1679	94.90%	T
H	1681	3.80%	A
H	1681	96.20%	G

Site 2: HNF3(2)-Sp1(1): nts 1713-1740

Genotype	Position	Variant Frequency	Variant Nucleotide(s)
A	1740	66.20%	C
A	1740	33.80%	T
A	1739	1.10%	A
A	1739	98.80%	G
A	1727	33.30%	A
A	1727	66.70%	G
A	1721	2.00%	A
A	1721	98.00%	G
A	1719	99.00%	G
A	1719	0.70%	T
A	1717	1.20%	A
A	1717	98.60%	G
B	1740	4.30%	C
B	1740	95.30%	T
B	1739	1.80%	A
B	1739	98.20%	G

B	1738	97.50%	A
B	1738	2.50%	T
B	1730	0.60%	A
B	1730	98.90%	G
B	1727	0.50%	A
B	1727	1.90%	C
B	1727	3.20%	G
B	1727	94.40%	T
B	1726	38.50%	A
B	1726	61.30%	C
B	1725	98.70%	A
B	1725	1.20%	G
B	1724	0.80%	C
B	1724	99.10%	T
B	1721	1.80%	A
B	1721	98.20%	G
B	1719	0.60%	C
B	1719	98.60%	G
B	1719	0.70%	T
C	1740	1.00%	C
C	1740	98.90%	T
C	1739	0.50%	A
C	1739	99.30%	G
C	1730	0.70%	A
C	1730	99.30%	C
C	1728	1.00%	A
C	1728	99.00%	G
C	1727	57.60%	A
C	1727	42.20%	G
C	1726	99.00%	A
C	1726	0.90%	C
C	1724	1.20%	C
C	1724	98.70%	T
C	1721	22.80%	A
C	1721	77.20%	G
C	1719	45.60%	G
C	1719	54.30%	T
D	1740	0.60%	C
D	1740	99.40%	T
D	1739	0.50%	A
D	1739	97.80%	G

D	1739	1.70%	T
D	1730	0.90%	A
D	1730	98.50%	C
D	1728	1.10%	A
D	1728	98.90%	G
D	1727	78.20%	A
D	1727	21.70%	G
D	1724	5.40%	C
D	1724	94.60%	T
D	1719	2.40%	C
D	1719	0.50%	G
D	1719	97.00%	T
E	1727	92.20%	A
E	1727	7.80%	G
E	1719	1.20%	G
E	1719	98.80%	T
E	1718	1.20%	C
E	1718	98.80%	T
F	1740	66.10%	C
F	1740	33.90%	T
F	1727	21.00%	A
F	1727	78.60%	G
F	1726	98.60%	A
F	1726	1.40%	C
F	1721	94.80%	A
F	1721	5.20%	G
G	1739	94.90%	A
G	1739	5.10%	G
G	1736	94.90%	A
G	1736	5.10%	G
G	1730	5.10%	C
G	1730	94.90%	G
G	1727	5.10%	A
G	1727	94.90%	T
G	1726	5.10%	A
G	1726	94.90%	C
G	1725	5.10%	A
G	1725	94.90%	G
G	1721	2.60%	A
G	1721	5.10%	G
G	1721	92.30%	T

H	1721	88.50%	A
H	1721	11.50%	G

Sp1(2): nts 1743-1752

Genotype	Position	Variant Frequency	Variant Nucleotide(s)
B	1752	53.60%	A
B	1752	2.40%	C
B	1752	42.10%	G
B	1752	1.80%	T
B	1751	1.90%	A
B	1751	97.70%	G
B	1750	98.90%	A
B	1750	0.90%	T
B	1748	1.10%	A
B	1748	98.80%	G
B	1747	99.10%	A
B	1747	0.60%	C
C	1752	96.90%	A
C	1752	2.50%	T
D	1752	83.80%	A
D	1752	13.10%	C
D	1752	2.80%	T
E	1752	95.50%	A
E	1752	4.50%	C
F	1745	98.60%	G
F	1745	1.20%	T
G	1752	5.10%	A
G	1752	94.90%	T
G	1748	5.10%	G
G	1748	94.90%	T
G	1746	94.90%	A
G	1746	5.10%	G
G	1745	94.90%	C
G	1745	5.10%	G
H	1752	11.50%	C
H	1752	88.50%	T

TBP-LSF/HNF4(2)-TBP: nts 1758-1776

Genotype	Position	Variant Frequency	Variant Nucleotide(s)

A	1773	1.60%	C
A	1773	98.40%	T
A	1768	2.90%	A
A	1768	0.70%	G
A	1768	96.10%	T
A	1766	93.30%	C
A	1766	6.10%	T
A	1764	30.60%	A
A	1764	69.10%	G
A	1762	71.30%	A
A	1762	28.50%	T
A	1758	1.10%	C
A	1758	98.80%	T
B	1775	96.40%	A
B	1775	3.10%	G
B	1773	88.40%	C
B	1773	11.60%	T
B	1768	2.30%	A
B	1768	97.60%	T
B	1766	97.60%	C
B	1766	2.30%	T
B	1764	16.00%	A
B	1764	84.00%	G
B	1762	83.70%	A
B	1762	16.20%	T
B	1760	99.10%	A
B	1760	0.80%	G
C	1775	79.40%	A
C	1775	0.50%	C
C	1775	20.10%	G
C	1773	97.90%	C
C	1773	1.70%	T
C	1768	3.10%	A
C	1768	96.60%	T
C	1766	94.80%	C
C	1766	5.00%	T
C	1764	50.90%	A
C	1764	48.90%	G
C	1762	51.90%	A
C	1762	47.90%	T
C	1760	97.00%	A

C	1760	2.80%	G
C	1758	6.00%	C
C	1758	93.20%	T
D	1775	98.00%	A
D	1775	0.50%	C
D	1775	1.50%	G
D	1773	60.20%	C
D	1773	39.70%	T
D	1772	98.30%	A
D	1772	0.60%	G
D	1772	0.60%	T
D	1768	2.60%	A
D	1768	97.20%	T
D	1766	0.80%	A
D	1766	85.90%	C
D	1766	9.70%	G
D	1766	3.50%	T
D	1765	0.60%	G
D	1765	99.10%	T
D	1764	25.10%	A
D	1764	0.70%	C
D	1764	66.80%	G
D	1764	7.40%	T
D	1762	76.10%	A
D	1762	23.30%	T
D	1761	93.40%	A
D	1761	6.10%	C
D	1758	1.20%	C
D	1758	98.80%	T
E	1775	98.00%	A
E	1775	1.60%	C
E	1773	98.80%	C
E	1773	1.20%	T
E	1772	98.80%	A
E	1772	0.80%	C
E	1769	0.80%	A
E	1769	99.20%	T
E	1768	96.50%	C
E	1768	3.10%	G
E	1764	12.20%	A
E	1764	87.50%	G

E	1762	87.10%	A
E	1762	0.80%	C
E	1762	11.80%	T
E	1761	97.30%	A
E	1761	2.40%	C
E	1760	99.20%	A
E	1760	0.80%	G
E	1759	2.40%	C
E	1759	97.60%	T
F	1773	0.60%	A
F	1773	42.50%	C
F	1773	56.90%	T
F	1768	3.60%	A
F	1768	96.40%	T
F	1766	96.40%	C
F	1766	3.60%	T
F	1765	0.80%	C
F	1765	99.20%	T
F	1764	24.40%	A
F	1764	75.60%	G
F	1762	76.00%	A
F	1762	24.00%	T
G	1775	97.40%	A
G	1775	2.60%	G
G	1773	5.10%	C
G	1773	94.90%	T
G	1768	2.60%	A
G	1768	97.40%	T
G	1765	92.30%	C
G	1765	7.70%	T
G	1764	97.40%	A
G	1764	2.60%	G
G	1762	2.60%	A
G	1762	97.40%	T
H	1773	3.80%	C
H	1773	96.20%	T
H	1768	3.80%	A
H	1768	96.20%	T
H	1766	96.20%	C
H	1766	3.80%	T
H	1764	3.80%	A

H	1764	96.20%	G
H	1762	92.30%	A
H	1762	7.70%	T

TBP (3): nts 1788-1795

Genotype	Position	Variant Frequency	Variant Nucleotide(s)
A	1790	13.00%	G
A	1790	86.80%	T
G	1795	2.60%	G
G	1795	97.40%	T
