# Supplementary figures and images for: Comprehensive Analysis of Hepatitis B Virus Promoter Region Mutations
Source: Viruses. 2018 Nov 1;10(11):603. doi: 10.3390/v10110603 (PMC6265984; doi:10.3390/v10110603)

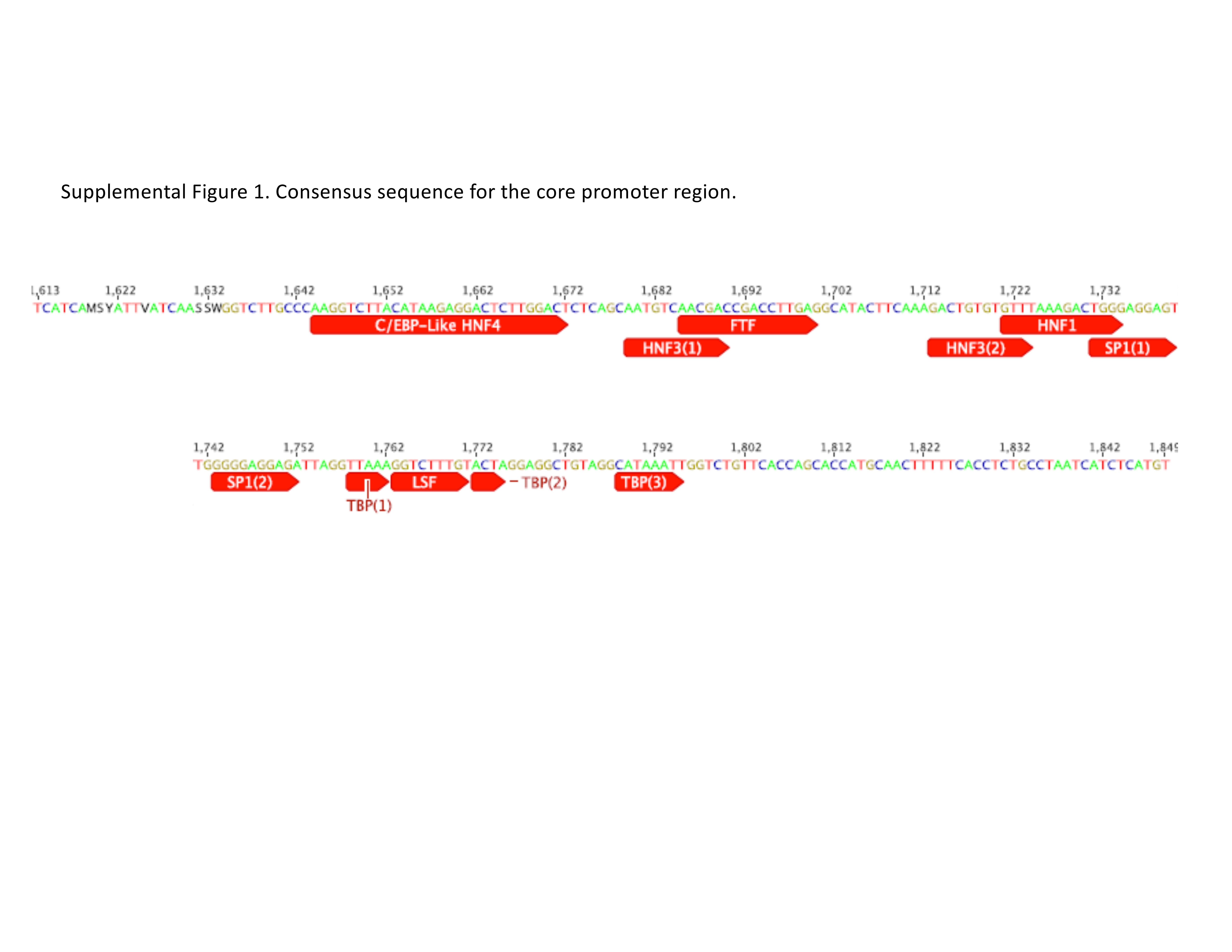

Supplement: Supplementary file 1 [file viruses-10-00603-s001.zip › viruses-380141-SI/viruses-380141-supplementary-FINAL/Viruses-380141-HBVpromoters-Figure_S1.Jpeg]
